# A Dermatological Dilemma: The Importance of Recognizing Dermatologic Manifestations of Drug Reaction With Eosinophilia and Systemic Symptoms (DRESS) in Skin of Color Patients

**DOI:** 10.7759/cureus.64061

**Published:** 2024-07-08

**Authors:** Nadim A Qadir, Christopher Marsalisi, Anvit D Reddy, Luke Stachler, Nirmal Onteddu

**Affiliations:** 1 Internal Medicine, University of Florida College of Medicine – Jacksonville, Jacksonville, USA

**Keywords:** drug reaction with eosinophilia and systemic symptoms, african american, skin of color, drug induced hypersensitivity syndrome, dermatology, vancomycin-induced dress syndrome, dress

## Abstract

Many dermatologic conditions that are seen in medical literature are typically on lighter skin tones making it easier to identify. This can pose a difficult problem in the care of skin of color patients. The purpose of this paper is to highlight the importance of dermatologic manifestations in skin of color patients and the disparities that exist in the medical field. Here, we present the case of a 51-year-old African American male who was hospitalized on a prolonged course of antibiotics found to have drug reaction with eosinophilia and systemic symptoms (DRESS). Although the initial diagnosis was not made at symptom onset due to the atypical presentation in darker skin tones, the patient improved when the diagnosis was eventually made with cessation of the offending agent and steroid therapy. There is a vital need for continued awareness of the disparities that exist within medical literature and the medical field in regard to skin of color patients.

## Introduction

Drug reaction with eosinophilia and systemic symptoms (DRESS), also known as drug-induced hypersensitivity syndrome (DIHS), is a rare and life-threatening reaction that presents with cutaneous manifestations generally 2-8 weeks after the offending agent's onset [1]. A dermatologic exanthem, eosinophilia, and organ damage characterize DRESS [2]. While the true incidence of DRESS is unknown, it is estimated at one in 1000 to one in 100,000 patients, occurring in mostly adults according to medical literature [1]. Although the pathophysiology is poorly understood, there is a well-reported association with medications such as anticonvulsants, sulfonamides, and antibiotics [3]. Here, we present the case of a 51-year-old African American male who was hospitalized for osteomyelitis and developed DRESS secondary to intravenous vancomycin. This case emphasizes the disparities in recognizing dermatologic manifestations of DRESS in individuals with darker skin complexion. Through the presentation of this case, we hope to raise awareness of this shortcoming and facilitate early recognition and diagnosis by clinicians in the future.

## Case presentation

**Figure 1:**
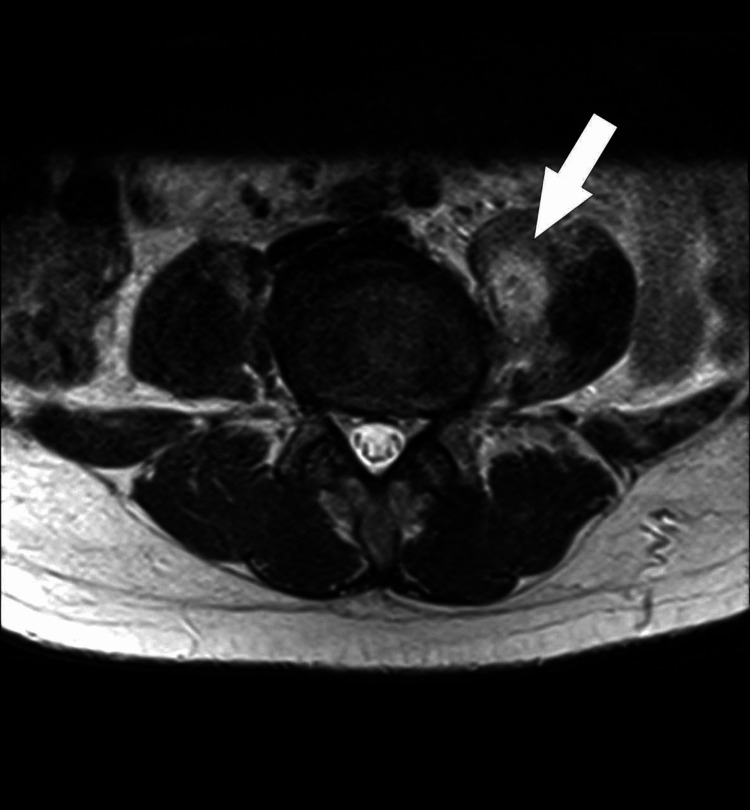
MRI axial T2-weighted sequence of the lower abdomen which reveals a small abscess within the left psoas (arrow), with adjacent muscular edema. There is also minimal muscular edema within the right psoas muscle. MRI: magnetic resonance imaging

**Figure 2:**
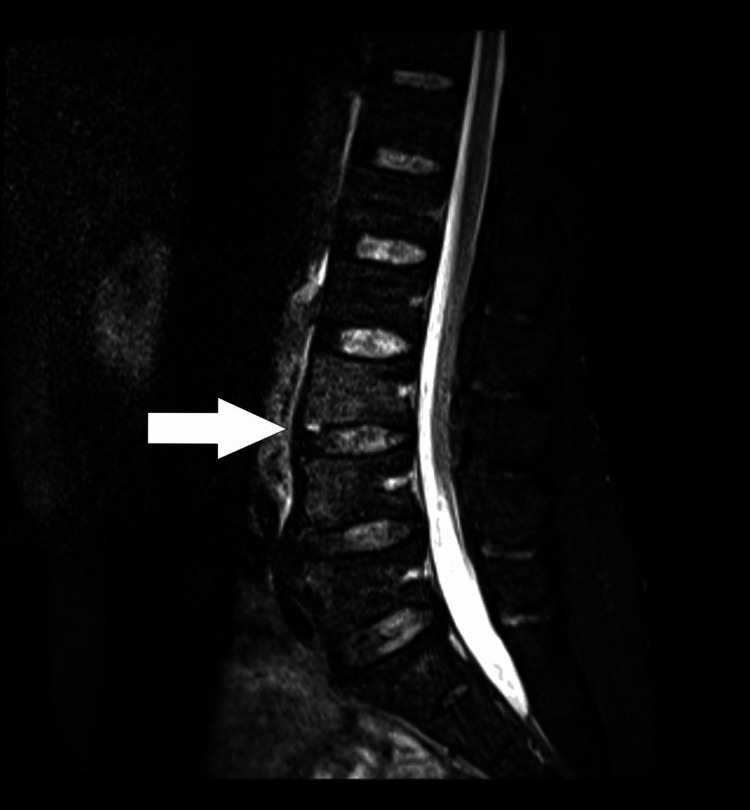
Sagittal MRI with STIR sequence demonstrating mild vertebral body edema at the L3 and L4 levels concerning for discitis-osteomyelitis (arrow). MRI: magnetic resonance imaging; STIR: short tau inversion recovery

A 51-year-old African American male with a past medical history of polysubstance use disorder (cocaine and marijuana) initially presented to the emergency department due to lumbar and right shoulder pain for three days. The patient endorsed intermittent fevers and chills and denied nausea, vomiting, recent weight loss, difficulty walking, numbness or tingling in his upper and lower extremities, bowel or bladder incontinence, or sick contacts. The initial physical exam was remarkable for decreased right shoulder abduction secondary to pain and point tenderness over the L4 spinous process. Laboratory studies were significant for leukocytosis and elevated erythrocyte sedimentation rate (ESR) and C-reactive protein (CRP). Due to concern for osteomyelitis, magnetic resonance imaging (MRI) of the pelvis and lumbar spine was ordered, which demonstrated myositis of the bilateral psoas muscles with micro-abscesses and osteomyelitis of L3-L4 (Figure 1, Figure 2). The patient was started on empiric antibiotics with intravenous vancomycin and cefepime and admitted to the internal medicine team.

Early in the patient's hospitalization, the interventional radiology (IR) team was consulted for micro-abscess drainage and bone biopsy of lumbar vertebrae. Both interventions were deferred due to the small size of fluid collections. On day 2 of hospitalization, the patient's blood cultures grew *Streptococcus pneumoniae*, and antibiotics were narrowed to ceftriaxone. On day 12, the patient's hospital course was complicated by *Pseudomonas *pneumonia, and ceftriaxone was transitioned to cefepime. The patient remained inpatient and was pending placement at a long-term acute care (LTAC) facility for antibiotic administration via a peripherally inserted central catheter.

**Figure 3:**
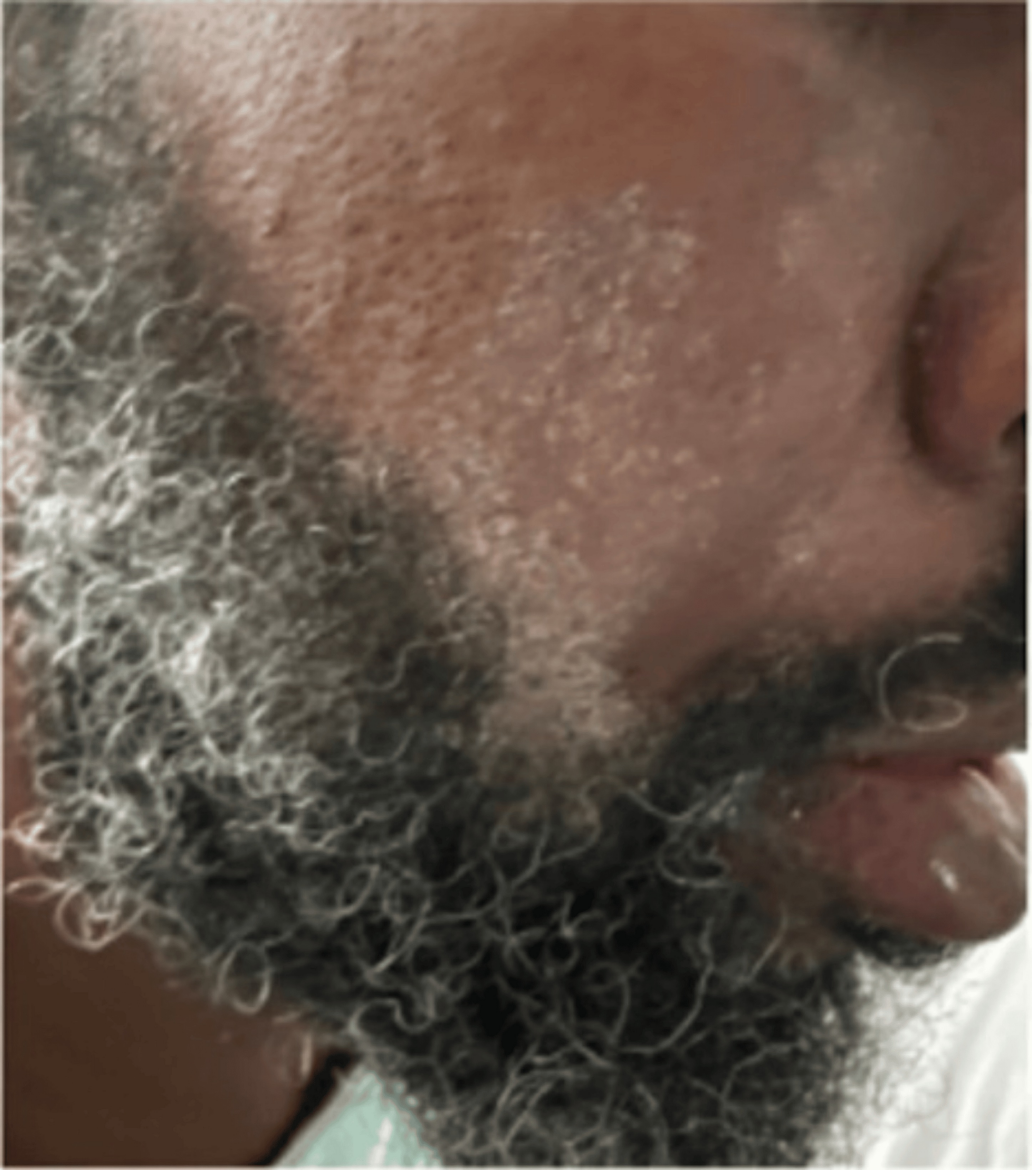
Coalescing white facial rash with skin sloughing.

**Figure 4:**
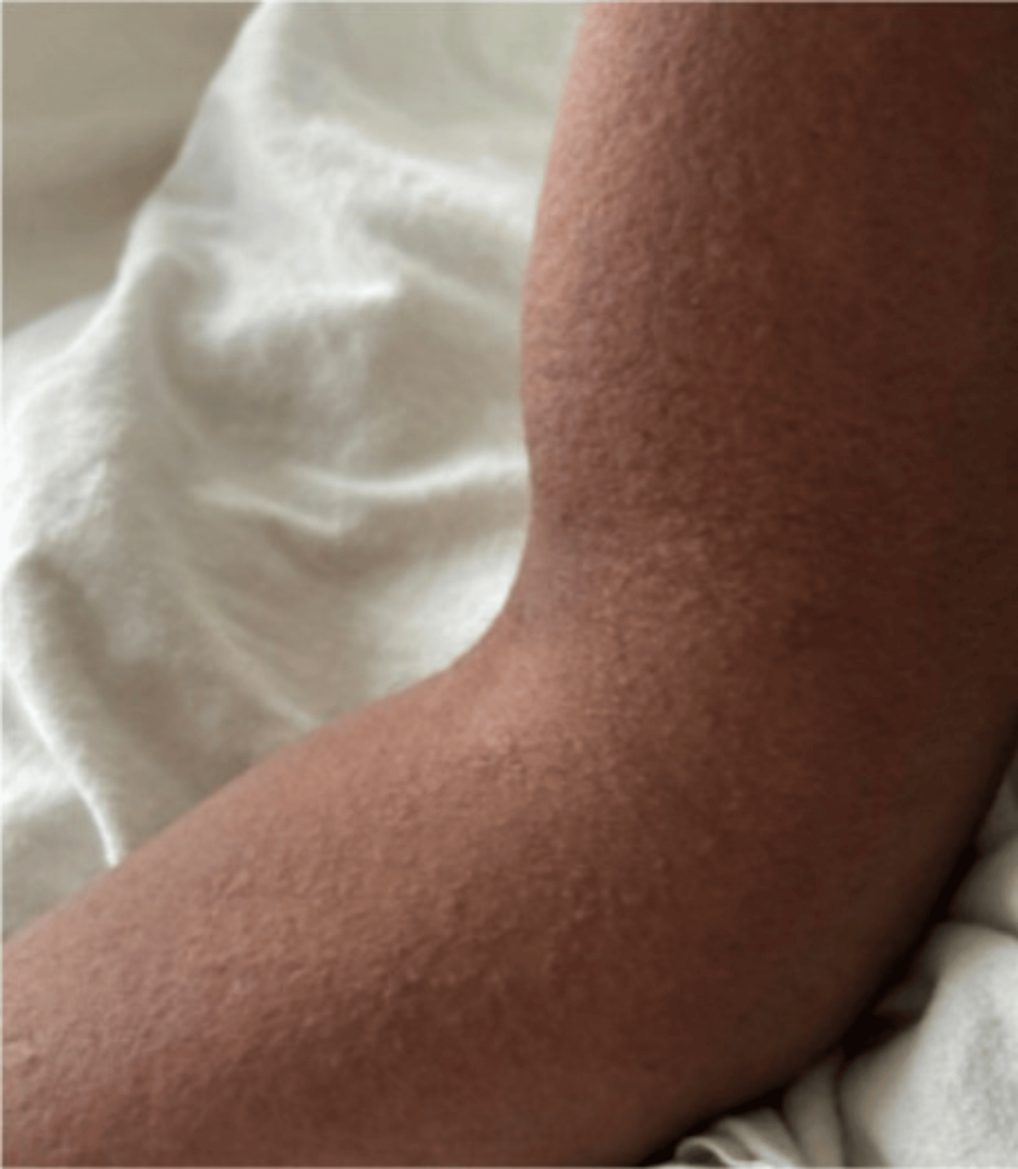
Morbilliform rash on the right upper extremity.

**Figure 5:**
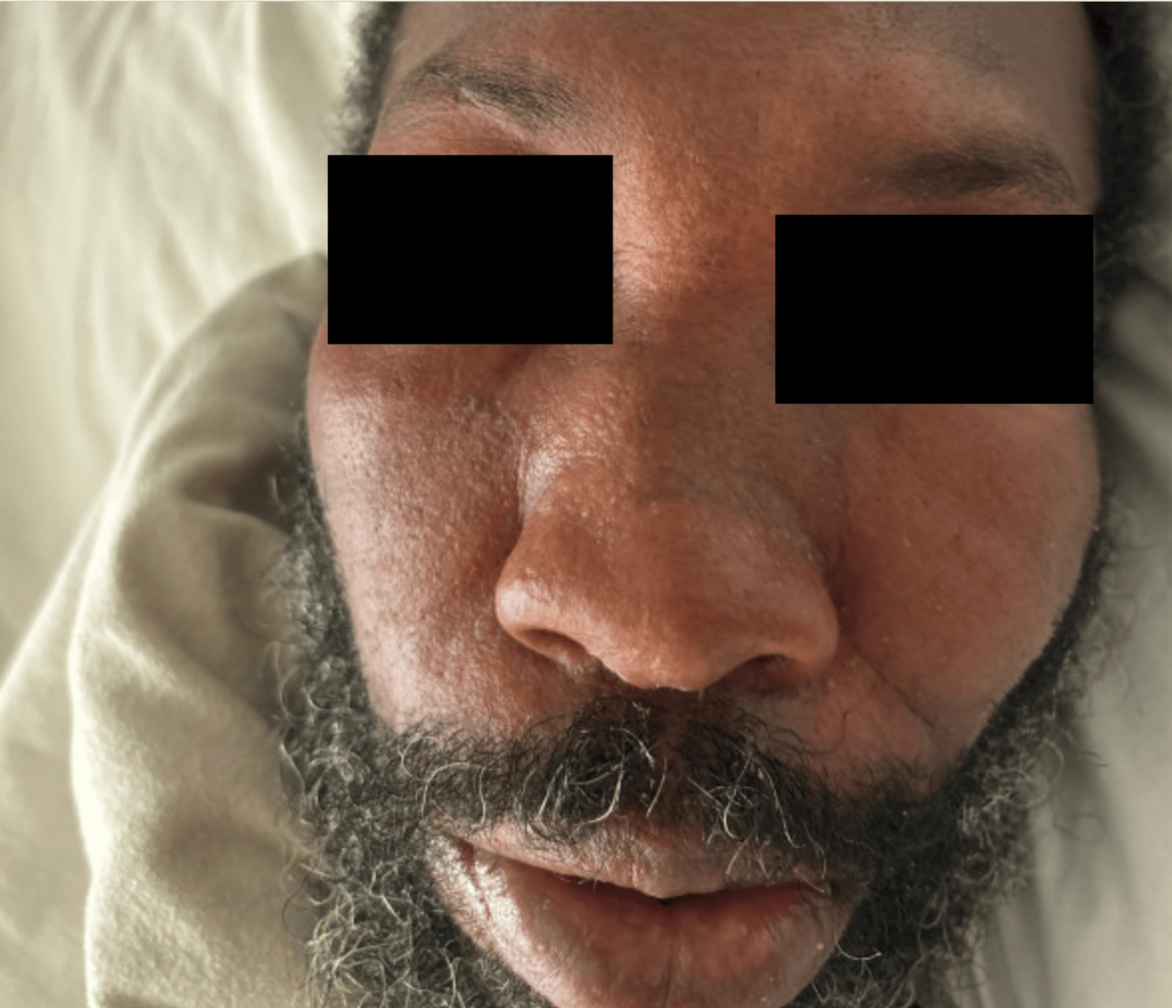
Periorbital and lip swelling seen.

The patient also had intermittent high-grade fevers with maximum temperatures of 39.4 degrees Celsius. On day 24, vancomycin was empirically added out of concern for concomitant infection. Two days later, the patient suddenly developed diffuse pruritus over his face, chest wall, and back, as well as bilateral upper and lower extremities. Additionally, white patches of skin and a morbilliform rash were noted diffusely along with axillary and inguinal lymphadenopathy (Figure 3, Figure 4). Laboratory findings at this time revealed uptrending leukocytosis, mild transaminitis, and elevated alkaline phosphatase (ALP). The right upper quadrant ultrasound and hepatitis panel were both negative. Repeat MRIs of the pelvis, lumbar spine, and right upper extremity were obtained which were unchanged from priors, and a repeat infectious workup was negative. The patient began experiencing episodes of diarrhea and cramping on day 27, with continued severe pruritus and worsening skin changes. Due to multiple bowel movements with continued uptrending white count at 18.35 THOU/CUMM, *Clostridium difficile* testing was obtained and found to be negative. Four days later, the patient then developed periorbital facial swelling bilaterally with no tongue swelling noted (Figure 5).

**Figure 6:**
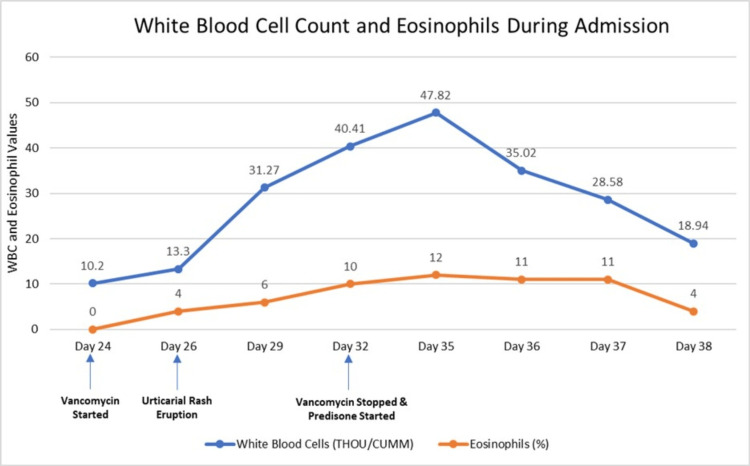
Line graph illustrating the trend of WBC count and eosinophils since offending agent onset. WBC: white blood cell

After much consideration, vancomycin was discontinued due to concern for DRESS syndrome in the setting of the aforementioned physical exam findings, uptrending leukocytosis with eosinophilic predominance, and transaminitis with elevated ALP. At this time, the infectious disease team was consulted and recommended transitioning antibiotics to daptomycin, and the patient was started on oral prednisone 0.5 mg/kg (day 32). In the following days, the patient experienced a drastic improvement in his rash, urticaria, and periorbital swelling, as well as the resolution of his leukocytosis and transaminitis (Table 1). Due to the temporal relationship between the initiation of prednisone and discontinuation of the likely agent with improvement of the patient's clinical picture, a diagnosis of DRESS was made (Figure 6). The patient was then sent to long-term care with a 10-week taper of prednisone and a four-week course of intravenous daptomycin to complete the antibiotic course. Upon detailed chart review, the patient has experienced a complete resolution of reported symptoms and has since been discharged from the LTAC facility.

**Table 1:** Hospital course laboratory value trends of liver enzymes, WBC count, eosinophils, and alkaline phosphatase in the setting of DRESS. DRESS: drug reaction with eosinophilia and systemic symptoms; AST: aspartate aminotransferase; ALT: alanine transaminase; WBC: white blood cell

	Reference range	Day 26	Day 29	Day 32	Day 35	Day 36	Day 37	Day 38
AST (IU/L)	14-33	156	93	71	146	101	81	102
ALT (IU/L)	10-42	260	219	156	169	193	173	178
WBC (THOU/CUMM)	4.5-11	13.3	31.27	40.41	47.82	35.02	28.58	18.94
Eosinophils (%)	0-5	4	6	10	12	11	11	4
ALP (IU/L)	40-129	476	686	542	358	370	339	299

## Discussion

DRESS is a relatively uncommon condition, affecting approximately one in 10,000 patients who receive an offending agent, and typically presents with a variety of symptoms 2-8 weeks after exposure [4]. Patients may present with non-specific symptoms such as rash, fever, lymphadenopathy, pruritus, and facial swelling [5]. The most common cutaneous findings are a maculopapular eruption in 85% of patients and facial edema, which is generally a more severe phenotype [6]. It is expected to develop organ damage due to DRESS, with the liver being the most common, with 80% of patients having elevated liver function tests, and with the second most affected organ being the kidneys [7]. Morbidity and mortality drastically increase with severe systemic symptoms [1].

The pathogenesis of DRESS is not fully elucidated, but it is postulated to be due to several mechanisms. Genetically susceptible patients are believed to undergo CD4+ and CD8+ T-cell activation after exposure to the offending drug, resulting in excess tumor necrosis factor (TNF), interferon (IFN)-gamma, and interleukin production, ultimately leading to hypereosinophilia [8]. Drug exposure may also lead to concurrent viral reactivation, most commonly human herpesvirus 6 (HHV-6) and cytomegalovirus (CMV), causing further T-cell activation and contributing to further cytokine excess and hypereosinophilia [9,10]. Moreover, patients exposed to high-risk drugs such as vancomycin, allopurinol, aromatic antiepileptic drugs, and trimethoprim-sulfamethoxazole are more likely to develop this condition. Notably, vancomycin, which is the cause of DRESS in our patient, is related to approximately two-thirds of DRESS cases [11].

In practice, the European Registry of Severe Cutaneous Adverse Reactions (RegiSCAR) to drugs and collection of biological samples can be used to determine the likelihood of DRESS syndrome. A score above 5 correlates with a "definite" diagnosis of DRESS [12]. In the presented case, the patient scored 6 on the RegiSCAR in the setting of fever, eosinophilia, atypical lymphocytes, lymphadenopathy, skin involvement (rash consistent with DRESS), and end-organ involvement. Despite this relatively straightforward scoring system, some confounding factors may make the diagnosis more difficult. Namely, skin complexion and the ability of practitioners to recognize the typical rash on darker individuals present a challenge not only to DRESS but also to a variety of dermatologic conditions.

In recent years, the lack of significant representation of dark skin tones within the dermatologic text has been identified as a culprit for this medical dilemma. Although the racial and ethnic diversity of the US population is increasing, medical textbooks have failed to mirror this shift. Moreover, a recent study published in 2018 unveiled that only 4.5% of images in general medicine textbooks depicted dark skin [12]. Moreover, when considering the African American population, recent literature has documented more advanced dermatologic conditions when compared to Whites. This has been attributed to a variety of causes, including education, as mentioned above, the diversity of patients involved in clinical trials, limitations of objective scoring systems, and structural racism within medicine [13].

As with other integumentary conditions, these racial disparities have also impacted individuals diagnosed with DRESS syndrome. Notably, African American patients have an increased mortality when diagnosed with DRESS syndrome compared to white patients. While the exact cause is uncertain, it is believed to be a product of the difficulty in identifying the distinct bright red maculopapular pruritic rash associated with DRESS syndrome [14].

The presented case was no exception to this statistic, with the patient's diagnosis being significantly delayed. The patient's symptoms were initially attributed to a recurrent infection as the classic DRESS dermatologic manifestations were challenging to identify. The aforementioned shortcomings within medicine related to integumentary conditions contributed to the delayed diagnosis in the presented case.

## Conclusions

DRESS is a delayed hypersensitivity reaction that can be easily missed in skin of color patients, which can further delay treatment. Treatment of this condition is to discontinue the offending agent, which can be difficult in itself as a number of medications have been associated with DRESS, and to initiate steroid therapy. It is imperative to treat early in the disease course as a delay in treatment can lead to significantly increased morbidity and mortality. There is a critical need for clinicians to recognize racial disparities in dermatological conditions such as DRESS to optimize skin of color patient outcomes.
